# An alternating breathing pattern significantly affects the brain functional connectivity and mood states

**DOI:** 10.3389/fnhum.2025.1539222

**Published:** 2025-04-16

**Authors:** Yulin Duan, Xun Guo, Bingnan Ren, Fang Liu, Yuhang Li, Fangfang Liu, Fan Xu, Min Huang

**Affiliations:** ^1^Department of Physiology, School of Basic Medical Sciences, Chengdu Medical College, Chengdu, China; ^2^Department of Clinic Medicine, School of Clinical Medicine, Chengdu Medical College, Chengdu, China; ^3^Department of Art, Art College, Southwest Minzu University, Chengdu, China; ^4^Department of Evidence-based Medicine and Social Medicine, School of Public Health, Chengdu Medical College, Chengdu, China

**Keywords:** functional connectivity, facial expressions, functional near-infrared spectroscopy (fNIRS), respiratory rehabilitation training, breathing pattern

## Abstract

**Introduction:**

To explore the impact of different breathing patterns on brain connectivity and emotional states.

**Methods:**

We recruited 31 participants with an average age of 19 years. They were instructed to perform controlled breathing, including calm, shallow, deep, and alternating deep and shallow breathing patterns. We employed functional near-infrared spectroscopy (fNIRS) to investigate disparities in the effects of multiple breathing patterns on the brain. Meanwhile, we captured the participants’ facial expressions and vital signs.

**Results:**

There were significant variations in the effects of four breathing patterns on functional connectivity between brain regions, facial expressions, and vital signs. The four breathing patterns impacted six brain regions. Among them, alternating deep and shallow breathing had a particularly pronounced effect, and there was robust functional connectivity in different brain regions. Additionally, this breathing pattern elevated autonomic nervous system activity, which contributed to achieving a more tranquil state. Furthermore, alternating deep and shallow breathing had a more positive influence on the changes in oxyhaemoglobin concentration (Δ [HbO_2_]) of the brain compared with deep breathing.

**Discussion:**

Alternating shallow and deep breathing could enhance emotional stability, improve autonomic nervous system function, and promote brain functional connectivity. Our findings unveiled distinct effects of diverse breathing patterns on both the brain and mood state, establishing a theoretical foundation for respiratory rehabilitation training for stroke patients.

## Introduction

Stroke continues to rank as the second leading cause of mortality globally and the third leading cause of both death and disability (Feigin et al., 2022). Stroke patients often present multi-system damage, among which the damage of the respiratory system manifests as respiratory dysfunction, including the weakening of respiratory function and muscle strength, as well as the weakening of diaphragmatic activity (Liaw et al., 2020). After a stroke, respiratory dysfunction frequently co-occurs with dysphagia, potentially increasing the risk of aspiration pneumonia. This complication represents one of the leading non-vascular causes of mortality following a stroke (GBD 2019 Stroke Collaborators, 2021). For stroke patients with this type of respiratory dysfunction, specialized rehabilitation training of the respiratory muscles typically yields significant benefits (Pollock et al., 2013). If movement disorders are present, intensive physical therapy combined with complementary therapies is frequently utilized to enhance cortical plasticity and achieve optimal functional outcomes (Johnstone et al., 2018). These approaches improve muscular strength in patients with neurological conditions and mitigates post-stroke complications (Menezes et al., 2016). Additionally, controlled breathing can modulate cortical excitability, thereby facilitating recovery in stroke patients with motor disorders (Kluger and Gross, 2020; Li et al., 2022).

The importance of respiratory rehabilitation training in stroke patients has attracted increased attention. Laborde et al. (2022) highlighted that slow breathing serves as an effective non-pharmacological intervention, significantly reducing heart rate and enhancing heart rate variability, which in turn improves cardiac health and autonomic nervous system function. These physiological benefits may also contribute to enhanced cognitive performance and emotional wellbeing. Jerath and Beveridge (2020) propose that respiratory rhythm can modulate the neurotransmitter balance and regional connectivity of the cerebral cortex by regulating autonomic nervous system activities, such as enhancing parasympathetic activity to promote relaxation or stimulating sympathetic activity to induce arousal. This regulatory mechanism not only influences emotional recognition and expression but also modulates cognitive functions like attention, memory, and decision-making through alterations in cortical excitability. Moreover, the combination of respiratory muscle training with inhalation and exhalation effectively enhances swallowing function, lung capacity, functional performance, and dysarthria in stroke patients (Wu et al., 2020; Zhang et al., 2022). Short-term inhalation and exhalation muscle training may also be a viable approach to improve respiratory muscle strength among stroke patients (Messaggi-Sartor et al., 2015). However, it is crucial to integrate these specific breathing patterns with tailored exercise programmes, educational interventions, and behavioral strategies to optimize overall health outcomes rather than solely focusing on symptom management (Rosenzweig et al., 2016).

Although existing respiratory training studies have provided preliminary insights into the influence of breathing patterns on physiological and psychological states, the precise mechanisms by which breathing directly affects brain activity, particularly in terms of emotion regulation and cognitive function, remain to be fully elucidated. To gain a deeper understanding of these complex interactions, this study employed two methodologies: functional near-infrared spectroscopy (fNIRS) and facial expression analysis.

The fNIRS technology enables non-invasive monitoring of cerebral cortex activity, particularly relevant for stroke patients, and has been validated as an effective tool for capturing neurophysiological changes associated with emotions and cognition. In previous studies, several works have explored the application of functional connectivity (FC) analysis in fNIRS. Yang et al. (2019) investigated neurodegenerative biomarkers in the prefrontal cortex of patients with mild cognitive impairment and assessed their functional connectivity using fNIRS. In another study, Yang et al. (2020) utilized FC strength derived from fNIRS to classify schizophrenia. These studies highlight the potential of FC analysis in fNIRS research and provide a foundation for subsequent investigations (Xia et al., 2022). Unlike prior studies that focused on general cognitive or emotional tasks, our research specifically examines the impact of breathing patterns on FC.

Concurrently, facial expression analysis, serving as a behavioral measurement method, offers valuable insights into the external manifestations and internal experiences of emotional states. Integrating these two approaches not only facilitates a multi-faceted evaluation of respiratory training effects but also sheds light on how breathing patterns influence emotions and cognition through modulation of cerebral cortex excitability and autonomic nervous system activity. Therefore, in this study, we employed functional near infrared spectroscopy (fNIRS) to record brain activity during experiments, while simultaneously capturing facial expressions through video analysis. This study revealed impacts of various breathing patterns on the brain and emotional states, laying a theoretical groundwork for respiratory rehabilitation programs to stroke patients.

## Materials and methods

### Inclusion and exclusion criteria

Thirty-one volunteers (12 men and 19 women) were recruited at Chengdu Medical College. They ranged in age from 18 to 21 years, with a mean age of 19.74. The inclusion criteria were: (1) the ability to cooperate with fNIRS experiments; (2) age ≥ 18 years old; (3) healthy without any ongoing medical conditions; and (4) agreed to participate in the study. The exclusion criteria were: (1) a history of severe or chronic respiratory disease; (2) a history of nervous system disease; (3) a history of mental illness; (4) a history of head surgery; and (5) other serious chronic diseases or undergoing other interventions.

### General workflow

It is imperative for researchers to ensure a serene laboratory environment to eliminate any potential sources of interference. The participants were assisted in donning the fNIRS system cap, while the video recording apparatus was positioned toward their faces. Once prepared, the researchers promptly initiated both the fNIRS signal detection system and video recording equipment. The participants then followed our self-designed programme prompts in E-prime to execute the entire experiment. This approach minimized errors as much as possible (Ogrinc et al., 2015).

**FIGURE 1:**
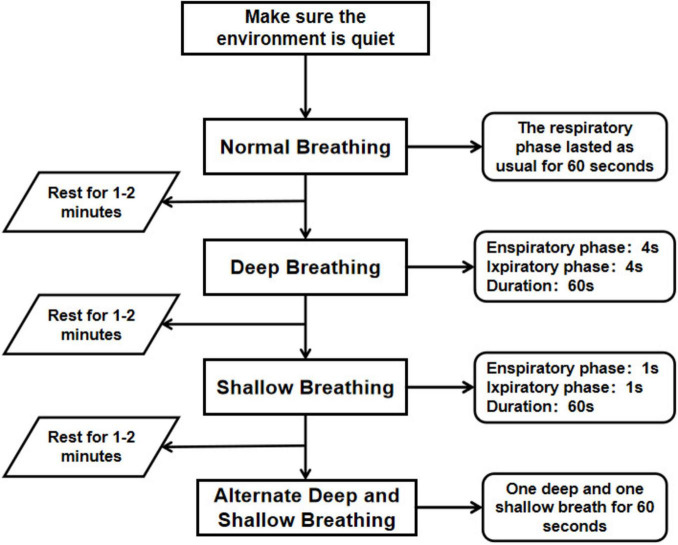
General experimental procedure.

The complete experiment comprised four distinct segments corresponding to different breathing patterns (Figure 1): calm breathing referred to the usual breathing rhythm; deep breathing consisted of a 4 s exhalation phase and a 4 s inhalation phase, making an 8 s cycle; shallow breathing consisted of a 1 s exhalation phase and a 1 s inhalation phase, making a 2 s breathing cycle; and alternating deep and shallow breathing involved alternating between deep breathing (4 s exhalation plus 4 s inhalation) and shallow breathing (1 s exhalation plus 1 s inhalation). Participants rested for 1–2 min while switching between the different breathing patterns.

### fNIRS data collection

Participants wore a 48-channel array consisting of 24 sources and 16 detectors with a source–detector separation of 3 cm (Supplementary Figure 1). The collected fNIRS data were categorized based on the breathing pattern. The relative concentrations presented in these data were obtained by measuring the amplitude attenuation of incident light and transmitted light with constant intensity, resulting in a discernible variation. A multi-channel continuous wave fNIRS system was used with two wavelengths of source light at 730 and 850 nm and a sampling rate of 11 Hz to measure changes in oxy- and deoxy-hemoglobin concentration (NirSmartII-3000A, Danyang Huichuang Medical Equipment Co., Ltd., China). It was selected for its ability to provide high-resolution spatial mapping, which is essential for accurately assessing the hemodynamic responses associated with different breathing patterns and their effects on specific brain regions. Furthermore, this system enhances the accuracy of source localization and signal quality in specific brain regions by precisely configuring the positions of source-detector pairs. It achieves this through advanced optical path design, rigorous data preprocessing, application of source-detector separation algorithms, spatial filtering, individualized calibration, and multi-channel recording techniques.

### Video data collection

The fNIRS system utilized in the experiment lacked the video recording capability. Experimental procedure of the subjects was recorded through the 2K intelligent camera system (Xiaomi, Beijing, China), and subsequently categorized based on the breathing pattern.

### fNIRS data pre-processing

For the preprocessing of fNIRS data, we adhered to a rigorous data processing protocol to ensure data integrity and reliability. This involved removing motion artifacts, reducing environmental noise, and normalizing the data to account for individual differences and device variability. All these procedures were conducted using NirSpark V1.8.1 software. The raw signals were denoised through the software’s denoising module to eliminate motion artifacts and physiological noises. Simultaneously, the signals were corrected to compensate for variations in light source intensity and differences in detector responses. The built-in algorithm was utilized to estimate the propagation path length of light through the scalp and brain tissue.

Spectral data with central wavelengths of 730 nm and 850 nm were selected and divided into four segments based on the sequence and duration of the breathing patterns. Each segmented portion resulted in a corresponding spark file, and all spark files were collected and categorized into four groups according to their respective breathing patterns.

Through the analysis of four groups of breathing pattern files, we obtained the values of total hemoglobin (Total Hb), deoxyhemoglobin (HbR), and oxyhemoglobin (HbO_2_) for each group. Since Total Hb measures the total amount of hemoglobin in cerebral blood vessels but cannot distinguish between oxygenated and deoxygenated forms, its sensitivity to changes in neural activity is limited (Akın, 2021; Ji et al., 2020). We chose HbO_2_ as the primary parameter because it is directly related to brain oxygenation and metabolic demands, which are crucial factors for assessing brain function. We converted the data into changes in oxyhemoglobin concentration [Δ(HbO_2_)] using the modified Beer-Lambert law. In addition, we calculated the cross-correlation coefficients between channels within each group to assess the functional connectivity of brain regions under each breathing pattern, and created functional connectivity maps using software (Li et al., 2015; Zondo et al., 2024).

### Video data pre-processing

The experimental videos underwent pre-processing through clipping. The four breathing patterns were extracted from the entire video. Any non-experimental processes within each video were eliminated to ensure that the analyzed video solely encompassed the breathing patterns of the subject.

Facial expression analysis was conducted using FacereaderV9.1 software. This software can recognize seven basic emotions: neutral, happiness, sadness, anger, fear, surprise, and disgust. During the analysis process, the software automatically detects faces in the video and marks facial feature points. It then calculates the intensity values for each emotion, which indicate the prominence of a particular expression in the video frames. It’s important to note that the accuracy of facial expression analysis can be influenced by lighting conditions during data collection. In environments with fluctuating lighting, the software’s performance may be compromised, potentially affecting the reliability of the results. To mitigate this issue, we ensured that environmental conditions were strictly controlled, particularly maintaining stable lighting to support accurate data collection and analysis. Additionally, while the software demonstrates advanced capabilities in detecting subtle expressions, extremely subtle or rapid facial movements may still pose challenges for complete and accurate capture.

### Vital sign analysis

The life signs (oxygen saturation levels, heart rate, heart rate variability, systolic blood pressure, and diastolic blood pressure) were monitored by the Mindray (VS-900) system.

### Data analysis

In this study, all data were initially subjected to a one-way ANOVA to determine if there were differences among the four breathing patterns. Further, a Turkey’s test was employed to ascertain if there were significant differences between groups. The statistical tests mentioned here, specifically for the analysis of brain region functional connectivity and Δ (HbO_2_), were conducted within the statistical analysis module of NirSparkV1.8.1 software. However, for facial expression and vital sign data, as the software used for extracting these data lacked analysis functionality, statistical analysis was performed using SPSS V27. A *P*-value < 0.05 was statistically significant.

## Results

### Functional connectivity

**FIGURE 2:**
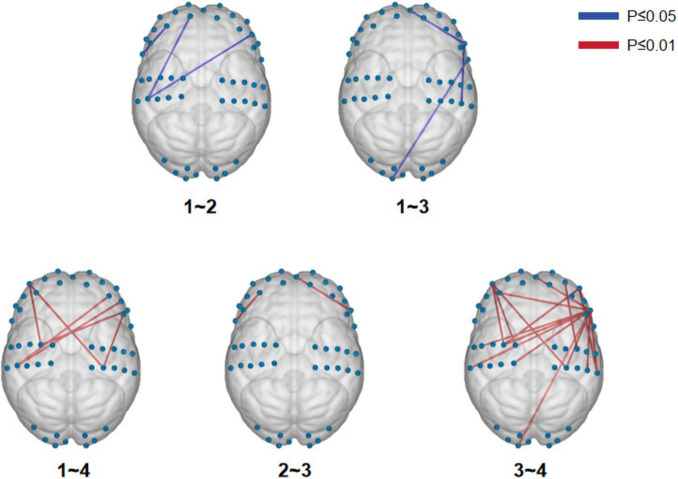
Multiple comparisons-visualization of functional connectivity differences in brain regions.

We initially conducted a one-way ANOVA to examine potential differences among the four breathing patterns. By utilizing the integrated statistical analysis function of NirSparkV1.8.1, we identified significant disparities in the functional connectivity of brain regions across 19 channels among four distinct breathing patterns (*P* ≤ 0.01), but these differences did not survive false discovery rate (FDR) correction (Supplementary Figure 2). Next, we proceeded with *post hoc* multiple comparisons to discern significant distinctions between each pair of breathing patterns. There were significant differences in functional connectivity between calm breathing and deep breathing, between calm breathing and shallow breathing, between calm breathing and alternating deep and shallow breathing, between deep breathing and shallow breathing, and between shallow breathing and alternating deep and shallow alternating (Figure 2 and Table 1).

**TABLE 1:** Results of multiple comparisons of functional connectivity in brain regions.

Channel name	1^1^	2^2^	3^3^	4^4^	*P*				
					**1∼2**	**1∼3**	**1∼4**	**2∼3**	**3∼4**
3∼14	0.214 (0.436)	0.422 (0.348)	0.193 (0.379)	0.531 (0.329)	0.13466	0.99608	0.00630	0.08196	0.00304
3∼16	0.259 (0.418)	0.348 (0.332)	0.186 (0.396)	0.526 (0.338)	0.78315	0.86561	0.02865	0.32022	0.00266
3∼20	0.406 (0.379)	0.448 (0.379)	0.122 (0.436)	0.515 (0.300)	0.97151	0.01923	0.66223	0.00504	0.00043
3∼24	0.335 (0.428)	0.421 (0.403)	0.177 (0.452)	0.520 (0.378)	0.85074	0.44398	0.30276	0.10325	0.00820
3∼27	0.395 (0.371)	0.450 (0.376)	0.204 (0.474)	0.574 (0.364)	0.94749	0.24196	0.29234	0.07758	0.00217
3∼32	0.396 (0.391)	0.451 (0.334)	0.267 (0.451)	0.584 (0.350)	0.94401	0.55074	0.22330	0.24177	0.00814
3∼34	0.428 (0.383)	0.524 (0.309)	0.307 (0.445)	0.612 (0.299)	0.72703	0.55389	0.19678	0.09140	0.00670
3∼35	0.381 (0.378)	0.360 (0.313)	0.109 (0.440)	0.489 (0.432)	0.99672	0.03717	0.70641	0.06343	0.00133
3∼41	0.249 (0.318)	0.277 (0.370)	0.028 (0.536)	0.410 (0.371)	0.99260	0.14829	0.40650	0.08056	0.00189
3∼44	0.141 (0.467)	0.241 (0.395)	0.020 (0.476)	0.383 (0.330)	0.78276	0.67245	0.11329	0.16890	0.00518
4∼41	0.286 (0.363)	0.196 (0.415)	−0.006 (0.553)	0.299 (0.373)	0.84472	0.04384	0.99950	0.26156	0.03267
5∼14	0.500 (0.318)	0.523 (0.383)	0.323 (0.384)	0.659 (0.266)	0.99334	0.17933	0.26612	0.10228	0.00102
5∼34	0.616 (0.299)	0.623 (0.270)	0.486 (0.354)	0.726 (0.175)	0.99963	0.27186	0.41495	0.22729	0.00574
7∼28	0.411 (0.343)	0.494 (0.340)	0.193 (0.467)	0.513 (0.382)	0.82998	0.12292	0.72344	0.01376	0.00762
9∼14	0.302 (0.384)	0.494 (0.403)	0.274 (0.414)	0.630 (0.293)	0.19258	0.99086	0.00459	0.10375	0.00169
9∼16	0.320 (0.339)	0.425 (0.351)	0.315 (0.339)	0.620 (0.275)	0.58520	0.99994	0.00245	0.55118	0.00204
9∼43	0.375 (0.300)	0.414 (0.419)	0.199 (0.438)	0.549 (0.315)	0.97707	0.24856	0.25999	0.11028	0.00180
12∼24	0.035 (0.478)	0.349 (0.431)	−0.003 (0.419)	0.233 (0.392)	0.02502	0.98593	0.27380	0.00913	0.14231
16∼18	0.329 (0.378)	0.478 (0.338)	0.472 (0.287)	0.606 (0.263)	0.25924	0.29308	0.00475	0.99987	0.35673
17∼33	0.311 (0.414)	0.555 (0.324)	0.460 (0.341)	0.619 (0.311)	0.03489	0.34182	0.00406	0.70883	0.28320
19∼33	0.194 (0.409)	0.366 (0.349)	0.335 (0.409)	0.529 (0.384)	0.31098	0.48451	0.00511	0.99005	0.20691
22∼33	0.308 (0.447)	0.591 (0.367)	0.459 (0.381)	0.523 (0.380)	0.02883	0.43764	0.14561	0.55897	0.91958

^1^Calm breathing group.

^2^Deep breathing group.

^3^Shallow breathing group.

^4^Alternate deep and shallow breathing group.

### Visualization of functional connectivity for each breathing pattern

**FIGURE 3:**
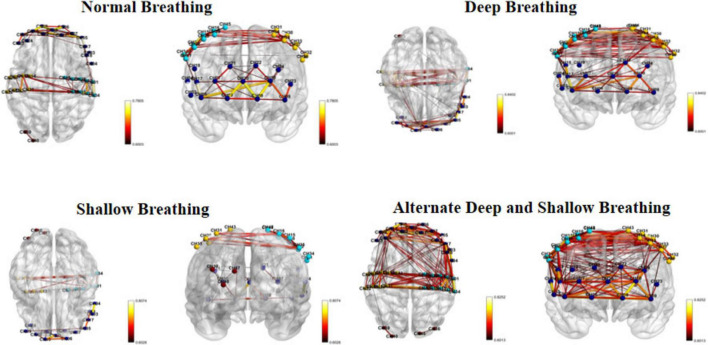
Visualization of channel functional links in brain regions.

Considering the notable variations in functional connectivity among the different brain regions during the four breathing patterns, we further visualized and compared the disparities in functional connectivity strength. To optimize the observation of brain functional connectivity maps for the four breathing patterns, an excessively high threshold may result in a reduced display of functional connections, while an overly low threshold may lead to an excessive display. Therefore, we selected 80 as the maximum threshold and calculated its 75th percentile value (0.60) as the threshold for this study. The selection of this threshold combination (a maximum threshold of 80 and the 75th percentile value) aims to strike a balance between sensitivity, which ensures the capture of as many relevant connections as possible, and specificity, which guarantees that only true functional connections are identified (Ren et al., 2022). Alternating deep and shallow breathing exhibited the strongest functional connectivity among the four breathing patterns (Figure 3).

**FIGURE 4:**
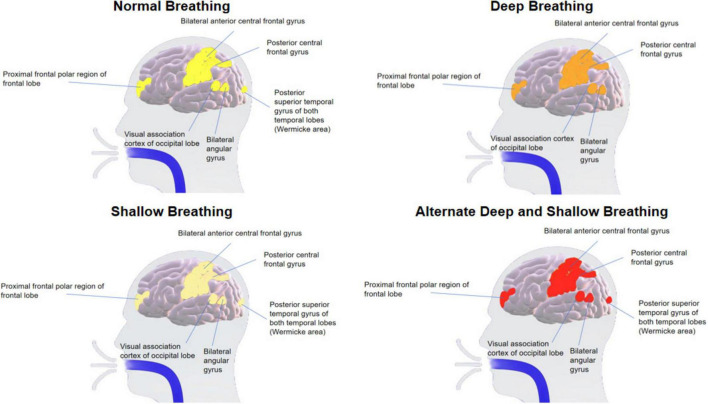
The Impact of Four Breathing Patterns on Brain Regions.

Based on the above results, we determined whether the different breathing patterns exerted distinct effects on brain regions through variations in functional connectivity channels. As illustrated in Figure 4, each breathing pattern impacted several brain regions, including the prefrontal pole of the frontal lobe, the bilateral central anterior gyrus of the frontal lobe, the bilateral central posterior gyrus of the parietal lobe, the visual association cortex of the occipital lobe, the bilateral angular gyrus, and the posterior part of temporal upper gyrus. Notably, alternating deep and shallow breathing exhibited the most robust influence on functional connectivity within these brain regions. Moreover, this pattern displayed a clear parietal lobe–frontal lobe–temporal lobe interconnection that was not observed with the other breathing patterns.

### Dynamic changes in Δ [HbO_2_]

**FIGURE 5:**
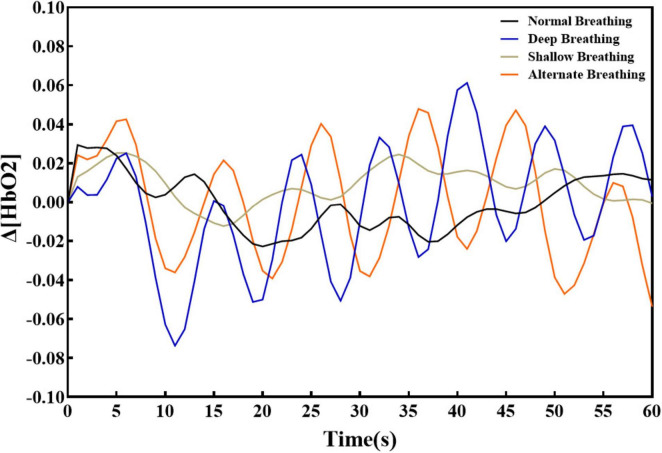
Dynamic changes of Δ [HbO_2_] in the four groups.

We analyzed the alteration of Δ [HbO_2_] by plotting the dynamic change curve (Figure 5) and a mean histogram (Supplementary Figure 3). Compared with deep breathing and alternating deep and shallow breathing, calm breathing led to smaller fluctuations in the Δ [HbO_2_] range and mean value. Additionally, shallow breathing produced a smaller fluctuation range, but a larger mean value compared with deep breathing, but the mean value was still smaller compared with alternating deep and shallow breathing. Alternating deep and shallow breathing reduced fluctuations in the Δ [HbO_2_] and led to a higher mean value compared with deep breathing.

### Facial expressions

**FIGURE 6:**
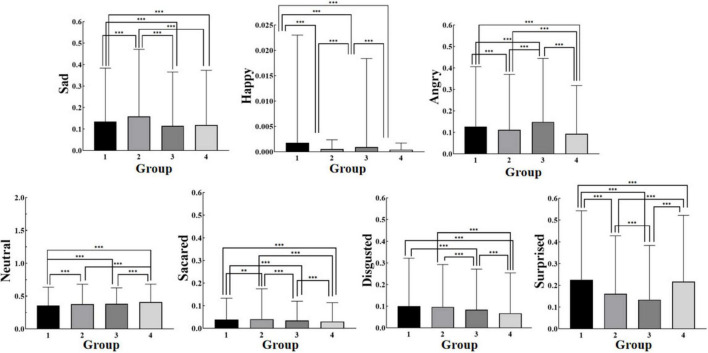
Facial expressions for each breathing pattern (Mean). * indicates a significant difference between the two groups for this emotion value at *P*≤0.05; ** indicates a very significant difference between the two groups for this emotion value at *P*≤0.01; *** indicates an extremely significant difference between the two groups for this emotion value at *P*≤0.001.

One-way ANOVA revealed significant differences in facial expressions among the four breathing patterns. The Turkey test for multiple comparisons indicated significant differences across all comparisons except for the neutral expression between the deep and shallow breathing patterns, happy between the deep and alternating deep and shallow breathing, and sad between the shallow and the alternating deep and shallow breathing patterns. Interestingly, alternating shallow and deep breathing produced the lowest levels of negative emotions (angry, sad, fear, hatred) and happy emotions. Moreover, this breathing pattern led to the highest level of the neutral expression along with minimal overall fluctuation range in emotional responses (Figure 6), suggesting that superior emotional control is associated with alternating deep and shallow breathing.

### Vital signs

One-way ANOVA revealed significant differences in vital signs among the four breathing patterns, except for systolic blood pressure. All vital signs across the four breathing patterns fell within normal ranges. Alternating shallow and deep breathing led to the highest standard deviation of the NN intervals (SDNN) and heart rate with minimal fluctuation, suggesting a more active autonomic nervous system and flexible heart rate regulation with this breathing pattern. Although blood oxygen saturation (SpO_2_) was comparatively lower with this breathing pattern, its fluctuation range remained small, indicating relatively stable blood oxygen regulation (Supplementary Figure 4).

## Discussion

Our research builds upon previous studies in the fields of fNIRS and cognitive rehabilitation. Similar Zondo et al.’s (2024) investigation into the effects of attention-based cognitive rehabilitation therapy on the behavior and hemodynamic responses of adolescent HIV patients, our work explores related themes (Zondo et al., 2024). Ji et al. (2020) utilized prefrontal-temporal fNIRS and functional connectivity based on seed regions to classify schizophrenia, while Akın (2021) proposed neurocognitive ratios derived from fNIRS as potential biomarkers for neuropsychiatric diseases. Additionally, Li et al. (2015) evaluated various diagnostic classification methods for schizophrenia using fNIRS. Although these studies primarily focused on diagnostic techniques, our research delves deeper into the potential influence mechanisms of breathing patterns on brain and cognitive functions. By integrating fNIRS with facial expression analysis, we aim to provide a comprehensive framework for assessing the effects of respiratory training and uncovering the intricate interactions among breathing patterns, brain activity, and cognitive function.

Breathing rehabilitation plays a pivotal role in the recovery process of stroke patients (Kang et al., 2022). By engaging in breathing rehabilitation, patients can effectively prevent complications, enhance ventilation function, facilitate lung recovery, improve quality of life, and promote holistic recovery (Al Chikhanie et al., 2021). This rehabilitative approach addresses common issues such as dysphagia and respiratory muscle weakness among stroke patients and proficiently maintains and fortifies respiratory muscle strength and lung capacity while mitigating the risk of respiratory system complications (He and Cheng, 2023). Consequently, this strategic intervention holds immense potential for optimizing outcomes in stroke patients. This study comprised a cohort of 12 men and 19 women. They were instructed to perform controlled breathing, including calm, shallow, deep, and alternating deep and shallow breathing patterns. We used fNIRS to investigate disparities in the effects of multiple breathing patterns on the brain, and we simultaneously captured the participants’ facial expressions and vital signs.

Distinct breathing patterns have been found to influence neural activity in diverse brain areas (Birn, 2012), to modulate various frequency ranges in brain dynamics (Birn et al., 2006), and to affect different biochemical (oxygen delivery and pH) and physiological variables (cerebral blood flow and heart rate variability) within the brain (Goheen et al., 2023). Our findings revealed that the four breathing patterns impacted several brain regions, namely the prefrontal pole of the frontal lobe, the bilateral central anterior gyrus of the frontal lobe, the bilateral central posterior gyrus of the parietal lobe, the visual association cortex of the occipital lobe, the bilateral angular gyrus, and the posterior part of temporal upper gyrus. Notably, among the four patterns, alternating deep and shallow breathing exerted the most robust influence on functional connectivity within these brain regions. Moreover, this pattern displayed a clear parietal lobe–frontal lobe–temporal lobe interconnection that was not observed with the other breathing patterns. In addition, there was less fluctuation and a higher Δ [HbO_2_] mean value compared to deep breathing. These results indicate that the corresponding brain regions were more active during alternating shallow and deep breathing.

Stroke patients often experience varying degrees of damage to different brain regions due to the location and severity of their hemorrhagic or ischemic lesions (Saver, 2006). Breathing exercises not only aid in the restoration of respiratory muscle strength and improvement in ventilation function for stroke patients (Fabero-Garrido et al., 2022), but also offer an effective approach to facilitate neural function reconstruction and to reestablish functional connections within the brain regions (Pascual-Leone et al., 2005). This study revealed that alternating deep and shallow breathing led to the most active functional connections across the four breathing patterns, suggesting that this breathing pattern may play a crucial role in reestablishing functional connections, thereby significantly enhancing patient prognosis and overall quality of life.

In addition, alternating deep and shallow breathing led to significantly lower scores for negative emotions (anger, sadness, and fear) and positive emotions compared with the other breathing patterns. Additionally, there were higher scores for the neutral expression along with minimal overall emotional fluctuations. These results suggest that alternating deep and shallow breathing exerts superior control over emotional states. Distinct breathing patterns have the potential to modify the impact of oscillations on memory and emotion processing (Courtney, 2009). Furthermore, extensive scientific literature supports the notion that respiratory techniques modulating rhythms and rates contribute to various psychological conditions such as depression, anxiety, post-traumatic stress disorder, among others (Brown and Gerbarg, 2005; Karavidas et al., 2007).

Vital signs include SDNN, an indicator utilized to assess heart rate variability by measuring the standard deviation of adjacent RR intervals (heartbeat intervals). It serves as a reflection of autonomic nervous system activity, enabling evaluation of the balance between cardiac sympathetic and parasympathetic nerves and determination of the presence or absence of heart rate variability (Sty and Stys, 1998). Alternating shallow and deep breathing led to significantly higher SDNN and heart rates with minimal fluctuations, this observation suggests that the autonomic nervous system activity of the deep-shallow alternating breathing group might exhibit greater dynamism compared to the other three groups.

In this study, we used fNIRS, vital signs, and facial expressions to monitor the physiological and emotional responses of the subjects. As an effective tool for monitoring brain function, fNIRS provides valuable data on cerebral oxygenation, revealing information about emotions, cognition, and neurophysiological activities. By combining the analysis of vital signs (such as heart rate and respiration) with facial expressions, we can enhance data interpretation and gain a multidimensional understanding of the subjects’ emotional changes and brain functions.

There were still some limitations. We did not include stroke patients in our study, and the sample size was limited. Furthermore, the inclusion of both male and female participants in this study without conducting separate gender analyses but rather employing mixed-gender analysis, along with the narrow focus on young individuals around 19 years old in terms of age, significantly restricts the generalizability of the research findings to other age groups. Hence, future investigations should aim to incorporate a larger sample size encompassing stroke patients from diverse age groups and genders, while also considering additional relevant variables to enhance the robustness and applicability of our research.

## Conclusion

Alternating shallow and deep breathing could enhance emotional stability, improve autonomic nervous system function, and promote brain functional connectivity. Our findings unveiled distinct effects of diverse breathing patterns on both the brain and mood state, establishing a theoretical foundation for respiratory rehabilitation training for stroke patients.

## Data Availability

The original contributions presented in this study are included in this article/Supplementary material, further inquiries can be directed to the corresponding authors.
