# Supplementary figures and images for: An alternating breathing pattern significantly affects the brain functional connectivity and mood states
Source: Front Hum Neurosci. 2025 Apr 16;19:1539222. doi: 10.3389/fnhum.2025.1539222 (PMC12040909; doi:10.3389/fnhum.2025.1539222)

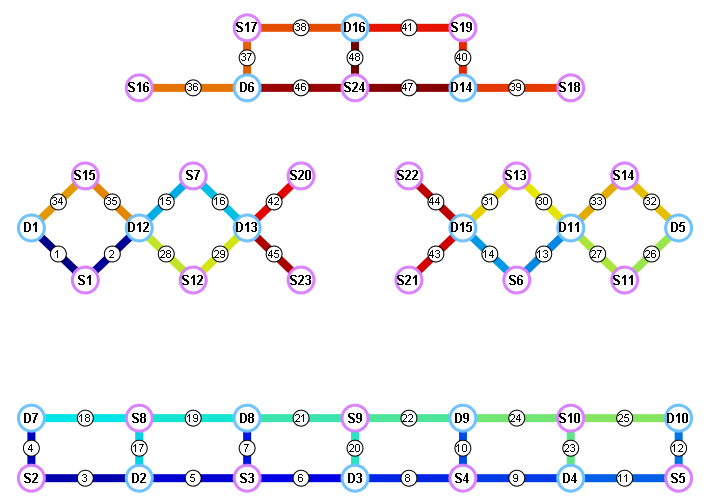

Supplement: Supplementary file 1 [file Image_1.tif]

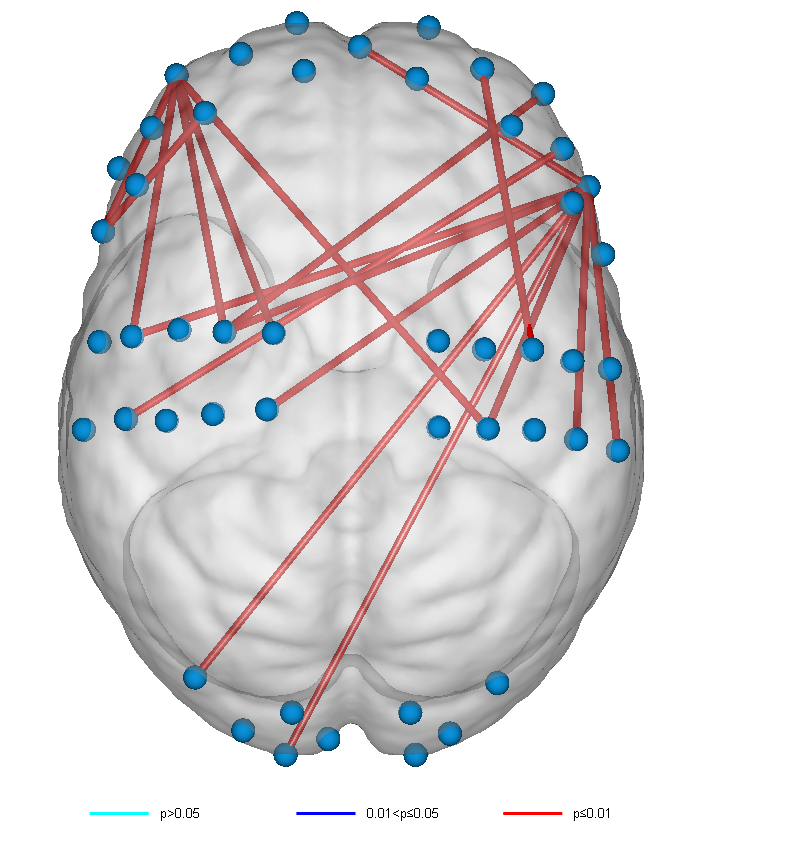

Supplement: Supplementary file 2 [file Image_2.tif]

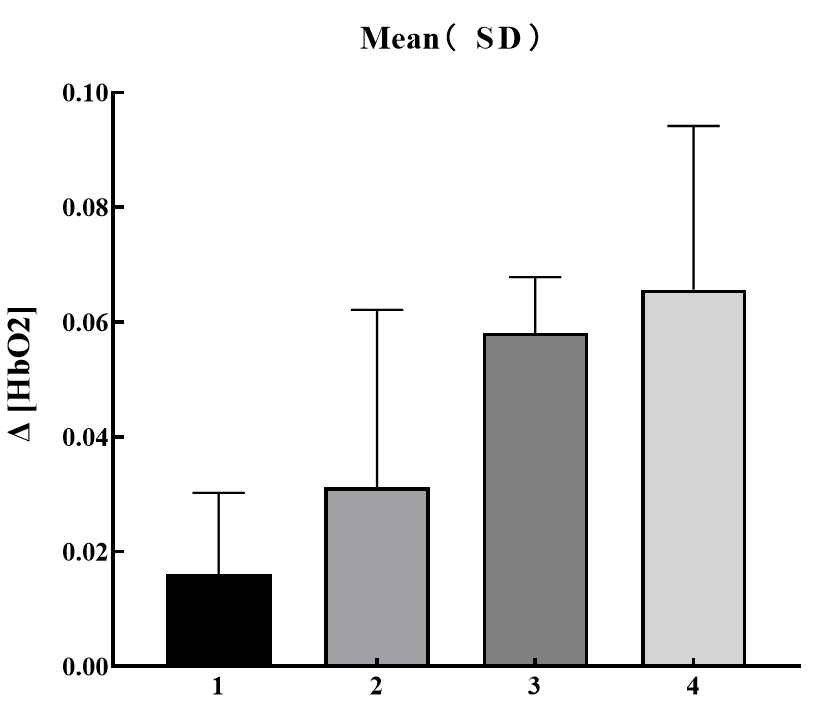

Supplement: Supplementary file 3 [file Image_3.tif]

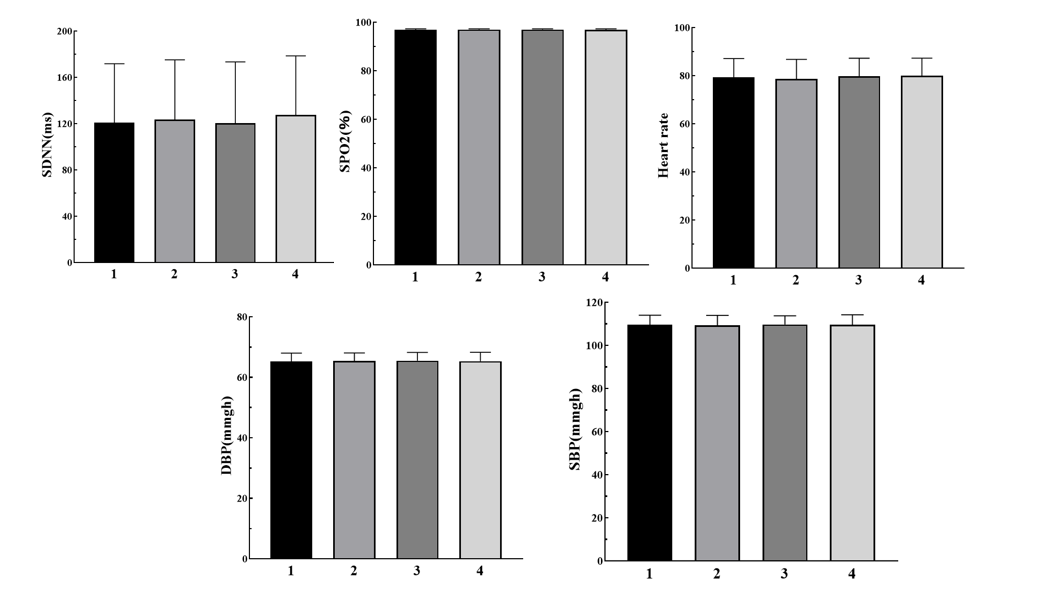

Supplement: Supplementary file 4 [file Image_4.tif]
